# Modelling of Molasses Fermentation for Bioethanol Production: A Comparative Investigation of Monod and Andrews Models Accuracy Assessment

**DOI:** 10.3390/biom9080308

**Published:** 2019-07-26

**Authors:** Hamid Zentou, Zurina Zainal Abidin, Robiah Yunus, Dayang Radiah Awang Biak, Mustapha Zouanti, Abdelkader Hassani

**Affiliations:** 1 Department of Chemical and Environmental Engineering, Universiti Putra Malaysia, Serdang 43400, Malaysia; 2 Department of Process Engineering, University of Djilali Bounaama, Khemis Miliana 44001, Algeria

**Keywords:** bioethanol, modelling, fermentation, molasses, Monod, Andrews

## Abstract

Modelling has recently become a key tool to promote the bioethanol industry and to optimise the fermentation process to be easily integrated into the industrial sector. In this context, this study aims at investigating the applicability of two mathematical models (Andrews and Monod) for molasses fermentation. The kinetics parameters for Monod and Andrews were estimated from experimental data using Matlab and OriginLab software. The models were simulated and compared with another set of experimental data that was not used for parameters’ estimation. The results of modelling showed that μ_max_ = 0.179 1/h and K_s_ = 11.37 g.L^−1^ for the Monod model, whereas μ_max_ = 0.508 1/h, K_s_ = 47.53 g.L^−1^ and K_i_ = 181.01 g.L^−1^ for the Andrews model, which are too close to the values reported in previous studies. The validation of both models showed that the Monod model is more suitable for batch fermentation modelling at a low concentration, where the highest R squared was observed at S_0_ = 75 g.L^−1^ with an R squared equal to 0.99956, 0.99954, and 0.99859 for the biomass, substrate, and product concentrations, respectively. In contrast, the Andrews model was more accurate at a high initial substrate concentration and the model data showed a good agreement compared to the experimental data of batch fermentation at S_0_ = 225 g.L^−1^, which was reflected in a high R squared with values 0.99795, 0.99903, and 0.99962 for the biomass, substrate, and product concentrations respectively.

## 1. Introduction

In the last century, an exponential increase in energy consumption and demand has been noted owing to industrial development, population and economic growth, and modernisation. Researchers have taken this seriously to fulfil future energy demand [1]. Currently, fossil fuel derived from non-sustainable energy sources is being used as the main source of power [2]. However, the high consumption of fossil fuels has led to certain economic and environmental implications owing to a depletion of fossil fuel reserves and global warming [3,4]. Renewable energy as biofuel, wind energy, solar energy, and hydroelectric energy can be alternate sources of sustainable power generation in order to replace conventional fossil fuel and to limit its implications. Renewable energy usage has increased in recent years, but it is not really widespread as it represents only 4.4% of the primary energy consumption [5]. The new renewable energy resources need to be developed, promoted, and supported for meeting the future needs of energy [6]. Among these renewable energy resources, biofuels such as bioethanol or biodiesel, which are predominantly produced from biomass, are the most promising alternate fuels owing to its advantages in terms of energy sustainability, reduction in the greenhouse effect, and rural areas development [7]. Starch and sugars are currently the main bioethanol production feedstocks, but there has been considerable debate about their sustainability in the recent years due to the competitivity between this sector and nutrition security, especially in the developing countries [8]. Thus, scientists have laid an emphasis on looking for new feedstocks for bioethanol production without implications on the agriculture sector and food security. In this regard, molasses has received more attention as it seems to be a promising feedstock for bioethanol production with a high yield and low cost and without competition to food crops [9]. Several researchers have highlighted the production of bioethanol using the alcoholic fermentation of molasses by *Saccharomyces cerevisiae* [9,10,11,12]. With the growing interest in the industrial application of batch alcoholic fermentation for bioethanol production, different kinetic models have been developed to describe microbial growth, product formation, and substrate consumption to serve the bioethanol industry. Owing to its help in the process control, a reduction in production costs, and an increase in the product quality, mathematical modelling may be regarded as a tool to optimise the fermentation process in order to meet the industry needs [13]. The mathematical modelling of fermentation processes can be classified into two main categories: structured and unstructured models. In unstructured models, the biomass is regarded as a chemical compound in a solution with an average formula, whereas it is regarded as a number of biochemical compounds in structured models taking into consideration the change in the internal composition of the organism [14]. Batch bioprocesses, in particular, are hard to model owing to the time-varying characteristics of biological systems, which often result in process nonlinearities. The multiplicity of reactions, the adaptability and evolution of organisms over short periods of time, and the continuous shift in environmental conditions are features that characterise batch processes. In this regard, a large number of studies have been conducted on the modelling of batch alcoholic fermentation kinetics. Batch reactors are firstly used to identify the main phenomena (limitation, inhibition, cell death, and maintenance, among others) that govern the fermentation kinetics by performing specific experiments for this purpose [15]. The literature related to the modelling of fermentation processes is quite extensive. However, the model presented by Monod (1950) seems to enjoy the widest acceptance model [16]. Table 1 reviews some recent articles highlighting the modelling of alcoholic fermentation using different feedstocks based on the Monod model and Andrews model.

The current study aims at developing and validating the Monod and Andrews mathematical models for predicting the dynamics of biomass, substrate, and ethanol for the batch fermentation of molasses using *Saccharomyces cerevisiae* besides assessing the accuracy of these models when compared with the experimental data different from the experimental data set used for the model development.

## 2. Materials and Methods

### 2.1. Microorganism and Fermentation Medium 

Three loops of active dry *Saccharomyces cerevisiae* yeast from Saf-Levure (Lesaffre, Marcq, France) were dissolved in 50 mL of distilled water, which were then added directly into 200 mL of culture media containing diluted molasses (50 g.L^−1^ glucose), ammonium sulphate (0.7 g.L^−1^), and ammonium phosphate (0.4 g.L^−1^). The fermentation medium was incubated at 35 °C besides shaking with 250 rpm for 6 h under aerobic conditions. All the used chemicals and materials were sterilized in an autoclave at 121 °C for 20 min before the experiment.

### 2.2. Fermentation

Anaerobic fermentation was carried out in a batch bioreactor with a 1-L volume containing 250 mL of culture media with different initial sugar concentrations (equivalent 5–25 g.L^−1^ glucose for the Monod model and equivalent of 50–200 g.L^−1^ glucose for the Andrews model), where the dilution rate was calculated in accordance with the chemical composition of molasses reported by Zentou et al. (2017) [9]. Yeast was added to the prepared fermentation medium with a concentration of 1 g.L^−1^ (calculated as fresh baker’s yeast). During the fermentation process, the pH value was adjusted at pH = 4.5 by the automatic addition of 0.1 M NaOH and the stirring speed was maintained at 250 rpm. The fermentation temperature was kept at 30 °C using a water jacket. The fermentation was carried out under micro-aeration conditions (1 vvm) for 2 h and turned later to anaerobic during the rest time of fermentation. A sample of 5 mL was taken at a predetermined time (0, 2, 4, 6, 8, 10, 12, 18, 24, 30, 36, 48, and 72 h) in order to determine the concentration of sugars, ethanol, and biomass.

### 2.3. Analytical Methods

Yeast growth was evaluated by spectrophotometric measurements at 620 nm in Shimadzu UV-1280 spectrophotometer (Shimadzu Scientific Instruments, Inc., Kyoto, Japan) and calibrated against the cell dry weight measurements. The concentrations of glucose, fructose, sucrose, and ethanol were measured by high performance liquid chromatography (HPLC) using a Biored Aminex HPX 87H column (Bio-Rad Laboratories, Inc., California, USA) as described in NREL (National Renewable Energy Laboratory, USA) methods [28]. In order to simplify the calculations, the concentration of other sugars (Fructose and sucrose) was converted into the equivalent concentration of glucose.

### 2.4. Models Theory 

#### 2.4.1. Growth Models 

In the Monod model, an emphasis has been laid to the exponential or logarithmic phase in modelling the microbial cells growth as the formation of the product is found maximum and directly proportional to the microbial cell growth. The growth rate of a batch culture under the exponential phase is generally believed to follow the first order kinetic model, i.e., the growth rate is proportional to the microbial mass in the system. Mathematically,

(1) $$\frac{dX}{dt}=µX$$

where $\frac{dX}{dt}$ denotes the bacterial growth rate (g.L^−1^.h^−1^), X (g.L^−1^) characterises the bacterial cell concentration, and µ (h^−1^) symbolises the proportional constant known as the specific growth rate. 

From Equation (1), the specific growth rate µ (h^−1^) can be written as follows:

(2) $$µ=\frac{\ln\left(x\right)-\ln\left(x_{0}\right)}{t-t_{0}}$$

The Monod equation describes the dependence of a microorganism’s growth rate on the concentration of a limiting substrate as follows:

(3) $$µ={µ}_{max}\frac{S}{K_{S}+S}$$

where µ_max_ is the maximum specific growth rate (h^−1^), *S* is the concentration of the growth limiting substrate (g.L^−1^), and K_s_ is the half velocity constant, i.e., the substrate concentration at half of the maximum growth rate (g.L^−1^). Combining Equations (1) and (3) yields:

(4) $$\frac{dX}{dt}=\frac{{µ}_{max}S}{K_{S}+S}\;X$$

The viable cell concentrations both in the carriers and the broth were determined and used for μ determination using Equation (2). The μ values were used to evaluate μ_max_ and K_s_ by Lineweaver–Burk plots derived from Equation (3). 

In the Andrews model, the growth of *S. cerevisiae* can be described in relation to the predicted inhibition caused by the excess of the substrate as follows:

(5) µ=µmaxSKS+S+(S2Ki)

The experimental growth data was fitted to determine the mathematical parameters (µ_max_, K_s_, and K_i_) using Origin 2018a software based on the SubstrateInhib function, where μ_max_ (h^−1^) is the maximum specific growth, K_s_ (g.L^−1^) is the half velocity constant, and K_i_ (g.L^−1^) is the substrate inhibition coefficient.

#### 2.4.2. Substrate and Product Models

Once μ_max_, K_s_ and K_i_ are calculated and μ is determined, the mathematical model for the substrate consumption and ethanol production can be expressed as follows:

(6) $$\frac{dS}{dt}=-\frac{1}{Y_{x/s}}\frac{dX}{dt}$$

(7) $$\frac{dP}{dt}=Y_{p/s}\frac{ds}{dt}$$

where Y_x/s_ and Y_p/s_ are the substrate- and product-specific yield coefficients, respectively.

### 2.5. Models Simulations and Validation 

The models were solved and simulated by using the fourth-order Runge–Kutta method ODE 45 with the MATLAB R2014a software (Version 8.3). The models’ performance was statistically estimated using the coefficient of determination (R^2^) where the simulation data were validated and compared with the experimental data set that was not used for parameters estimation. 

## 3. Results and Discussion 

### 3.1. Calculation of Kinetics Parameters

**Figure 1:**
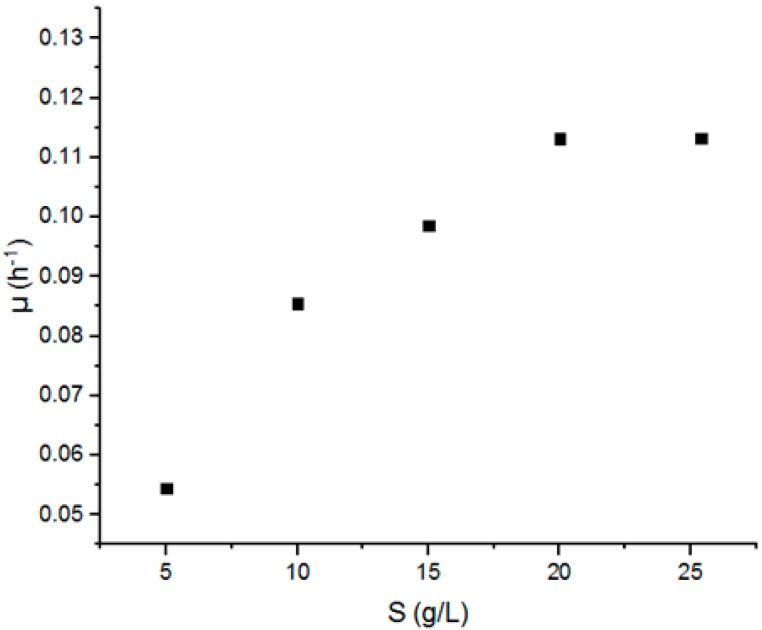
The variation of the specific growth rate μ as a function of the substrate concentration S (5–25 g.L^−1^).

In order to estimate the value of μ_max_ and K_s_ in the Monod model, a set of batch fermentation using different substrate (5–25 g) concentrations was carried out. Figure 1 represents the variation of specific growth coefficient μ as a function of substrate concentration. 

It is clear that the specific growth increases with the increase in the substrate concentration and that it achieved the maximum value for S = 20 g.L^−1^ and stabilized after that despite the augmentation of the initial sugar concentration.

μ_max_ and K_s_ were calculated based on Equation (3), which could be simplified to Equation (8):

(8) $$\frac{1}{\mu }=\frac{K_{S}}{\mu_{max}}\frac{1}{S}+\frac{1}{\mu_{max}}$$

**Figure 2:**
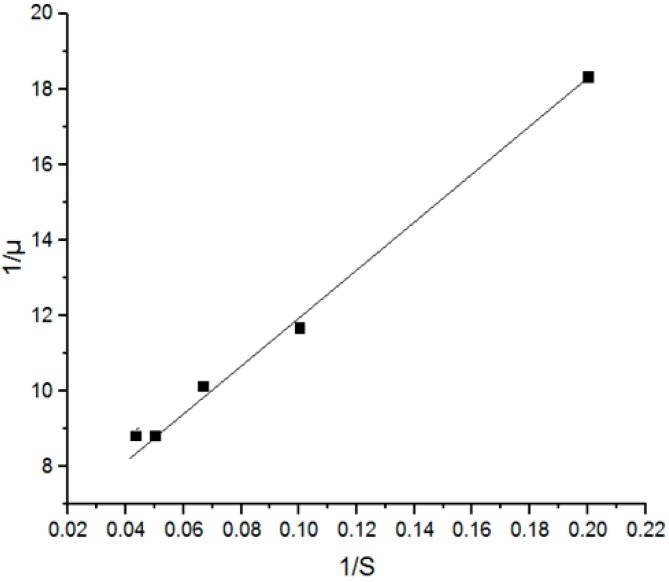
A Lineweaver–Burk plot estimating the μ_max_ and K_s_ values in a batch ethanol fermentation.

Figure 2 shows the Lineweaver–Burk plot estimating the μ_max_ and K_s_ values in a batch ethanol fermentation where the values of μ_max_ and K_s_ were found to be 0.179 h^−1^ and 11.37 g.L^−1^ respectively, which were too close to the values reported in previous studies [17,18] using sweet sorghum leaves and oil palm jus. However, these values were different from the values reported in other studies [19,20,21,22,23]. μ_max_ was 0.179 h^−1^, which is half of the value reported by Raposo et al. [23] for molasses fermentation, whereas K_s_ was almost double as compared to the same study and it amounted to 11.37 g.L^−1^. This difference may be due to the diversity of the used mediums and their composition, biomass concentration, and strain nature besides the change in operating conditions such as pH, temperature, and aeration which may affect the growth mechanisms. This confirms the need for a specific model for each feedstock fermentation separately, since it is not practical to generalize a standard model to be applicable to all sets of fermentation.

For the Andrews model, the mathematical model coefficients (µ_max_, K_s_, and K_i_) have been estimated for a set of batch fermentation at different substrate concentrations (100, 200, and 300 g.L^−1^) using origin 2018a software based on the SubstrateInhib function. 

**Figure 3:**
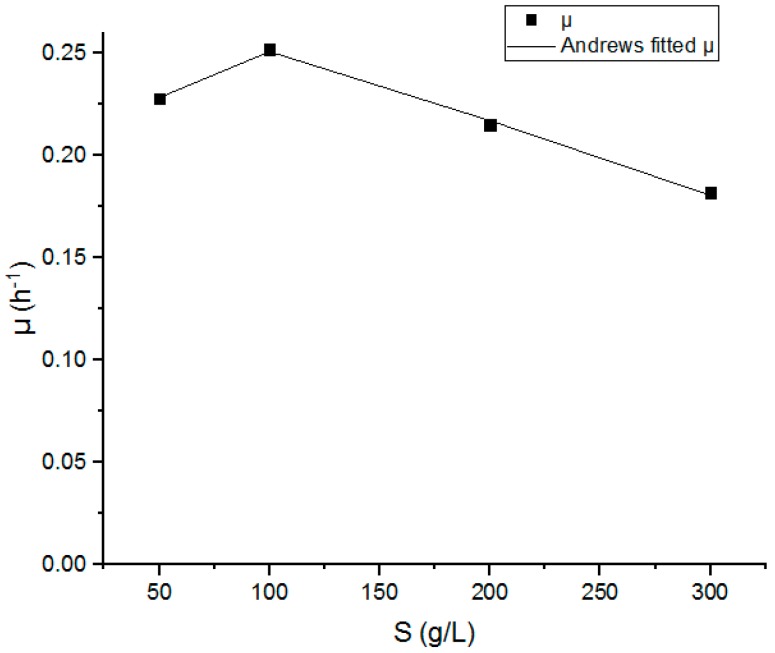
The variation of the specific growth rate μ as a function of the substrate concentration S (50–300 g.L^−1^).

Figure 3 represents the variations of the specific growth rate μ as a function of the initial substrate concentration S (50–300 g.L^−1^). The graph shows that the specific growth rate μ increased from 0.225 to 0.250 h^−1^ with the increase in the glucose concentration in the range of 50–100 g.L^−1^. However, a remarkable decline in the specific growth rate μ from 0.250 to 0.185 h^−1^ was noted at the range of 100–300 g.L^−1^ of the glucose concentration which is contrary to the reported theory in the Monod model. It confirms that the continuity of the specific growth rate increases with the increase in the glucose concentration.

The Andrews kinetics parameters were estimated using the origin 2018b Software and are presented in Table 2, where μ_max_, K_s_, and K_i_ were 0.508 h^−1^, 47.53 (g.L^−1^), and 181.02 (g.L^−1^), respectively, and were estimated with a high accuracy (R^2^ = 0.9907).

### 3.2. Calculation of Yield Coefficients

The formulation of Equations (9) and (10) are based on the parallel conversion stoichiometry equations. The cell mass yield coefficient (Y_x/s_) and the product yield coefficient (Y_p/s_) can be calculated during the growth phase as follows:

(9) $$Y_{x/s}=\frac{\Delta X}{\Delta s}$$

(10) $$Y_{p/s}=\frac{\Delta P}{\Delta s}$$

Table 3 represents the values of Y_x/s_ and Y_p/s_ respectively in the range between 5 and 25 g.L^−1^. No significant change in both yield coefficients was noted in this range, and the average values of Y_x/s_ and Y_p/s_ were 0.518 and 0.365, respectively. The same was noted for the Andrews model where the average values of Y_x/s_ and Y_p/s_ were 0.286 and 0.431 respectively.

### 3.3. Model Validation

To validate the Monod and Andrews models, the simulation data of the model was compared with the experimental data obtained from the batch fermentation with different initial substrate concentrations (75, 150, and 225 g.L^−1^). A common way to assess the reliability of the model is to use statistical indicators such as the coefficient of determination (R^2^). Although there is much discussion in the literature about the validity of using R^2^ to validate a nonlinear model, this statistic can provide a fair first indication of how much of the variance in the experimental data is explained by the model. However, a key limitation of R^2^ is that this statistic cannot determine whether the parameter estimates and predictions are biased, which is why the residual plots must be assessed. Despite this limitation, the value of R^2^ has been widely accepted to validate the mathematical models of bioprocesses [27,28,29,30,31].

**Figure 4:**
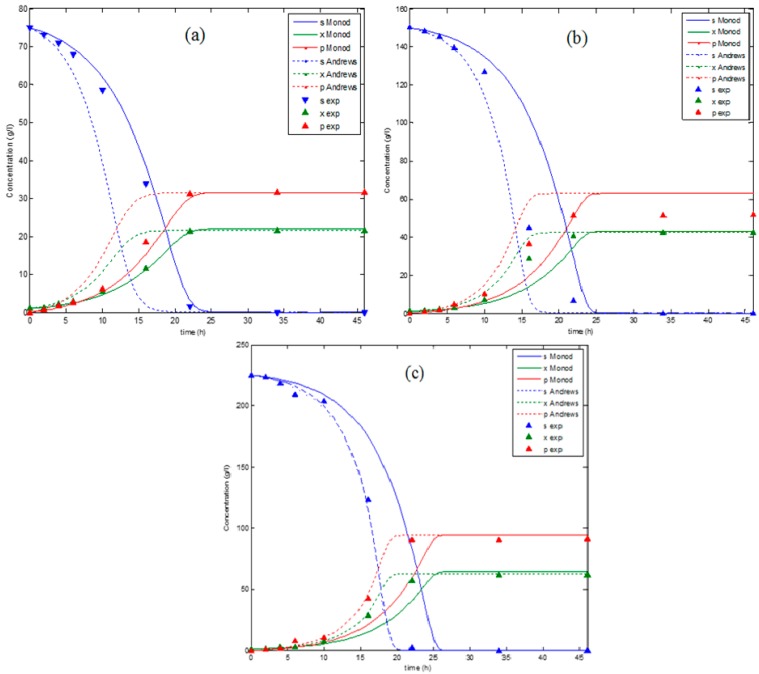
A comparison between the models’ data and the experimental data for x_0_ = 1 g.L^−1^ at different substrate concentrations: (**a**) S_0_ = 75 g.L^−1^, (**b**) S_0_ = 150 g.L^−1^, and (**c**) S_0_ = 225 g.L^−1^.

Figure 4 represents the simulation data using Matlab 2014a for the Andrews and Monod models at different substrate concentrations (75, 150, and 225 g.L^−1^) as compared to the experimental data of a series of batch fermentation at the same concentrations (75, 150, and 225 g.L^−1^). The values of substrate concentration used for the validation of the models are different from the concentrations which were used for the modelling which gives the validation more reliability. Figure 4 showed a variance in both models’ accuracy when they were compared to the experimental data. This variance seemed to be dependent to the initial substrate concentration. However, a statistical analysis is needed for more validation.

**Figure 5:**
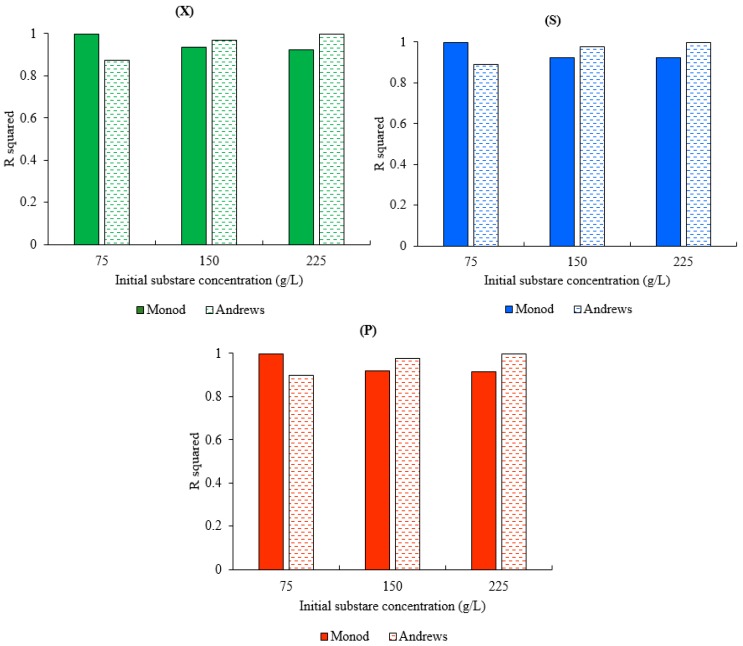
R^2^ of both models for x_0_ = 1 g.L^−1^ at different initial substrate concentrations: (**X**) R^2^ for the biomass concentration, (**S**) R^2^ for the substrate concentration, and (**P**) R^2^ for the ethanol concentration.

Figure 5 represents the variance of R^2^ of both models for biomass, substrate, and product concentrations at different initial substrate concentrations (75, 150, and 225 g.L^−1^). The results represented in Figure 5 show the dependency of both models’ accuracy on the initial substrate concentration. The Monod model showed a good agreement with the experimental data at a low concentration where the maximum R^2^ values for the biomass, substrate, and ethanol concentrations were achieved at S_0_ = 50 g.L^−1^. The decline in R^2^ of the Monod model was observed with the increase in the initial substrate concentration from R^2^ biomass = 0.99956, R2 substrate = 0.99954, and R^2^ product = 0.99859 to R^2^ biomass = 0.92462, R^2^ substrate = 0.921547, and R2 product = 0.916246 for an increase in the initial substrate concentration from 75 to 225 g.L^−1^. In contrast, the Andrews model showed a good performance at high concentration (225 g.L^−1^) with R^2^ equal to 0.99795, 0.99903, and 0.99962 for the biomass, substrate, and product concentrations, respectively. However, the Andrews model was less accurate at a low concentration, with R^2^ equal to 0.8743932, 0.891686, and 0.90020 for the biomass, substrate, and product concentrations, respectively at S_0_ = 75 g.L^−1^. It was also noted that any change in the R^2^ biomass led to a change in the R^2^ substrate and the R^2^ product in both models, which is understood due to the association of the product and substrate expressions with the cell growth expression in both models.

The low accuracy of the Monod model in a high substrate concentration is due to the fact that the classical Monod equation does not consider the inhibitory effect of a high substrate on cell growth. On the other hand, the Andrews model has successfully presented the experimental dataset at a high concentration; however, it does not show the same performance at a low concentration. This can be explained by the estimation of the Andrews model coefficients at high substrate concentrations in order to investigate the inhibitory effect of the substrate which leads to a low accuracy at low initial substrate concentrations as compared to the Monod model. These results confirm the limitation of these models to widely describe the fermentation process. However, these models can be more practical under specific conditions. Despite the efforts devoted to developing complex models to be more accurate and more practical, a universal model for different fermentation cultures does not exist. Although increasing the model complexity often results in an improved curve fitting, the most appropriate model should be selected on the basis of statistical considerations. Unfortunately, there is evidence that complex equations have often been constructed in an attempt to explain a set of experimental data that exhibited so much scattering that it was impossible to discriminate between the different models. Although both the numerical and analytical methods played a critical role for the advancement of these models, it will still be difficult to find the necessary balance between avoiding an unnecessary complexity and ensuring an adequate reality [32].

## 4. Conclusions

A mathematical model which predicts, simulates, and controls the alcoholic fermentation development would be a valuable technical tool to promote the bioethanol industry and to increase its economical competitivity. The Monod model is the most used basic model to simulate alcoholic fermentation. However, different modified models have been developed based on the Monod model to ameliorate the model reliability and acceptance. In the present study, a comparative investigation was conducted to evaluate the performance and reliability of the Monod and Andrews models for batch alcoholic fermentation. However, the results confirm that it is so difficult to define the most suitable model at different microbial growth conditions. However, the suitability of a model can only be determined for a specific range.

It was found that the Andrews model showed a better agreement with the experimental data at a high initial substrate concentration due to the consideration of the substrate inhibitory effect. On the other hand, the Andrews model was less accurate at a low initial substrate concentration compared to the Monod model which showed more suitability to represent the experimental data at a low concentration. It is clear that the complexity of a model does not always serve the objective to develop the accuracy of a model but can only be used under certain conditions when the simple models fail to give a real representation of the experimental data.

## Figures and Tables

**Table 1:** The fermentation kinetics parameters of the Monod model using different feedstocks.

Substrate	Model	µ_max_ (h^−1^)	K_s_ (g.L^−1^)	K_i_ (g.L^−1^)	Reference
Sorghum leaves	Monod	0.176	10.11	----	[17]
Oil palm frond juice	Monod	0.150	10.21	----	[18]
Sweet sorghum juice	Monod	0.313	47.51	----	[19]
Banana peels	Monod	1.500	25.00	----	[20]
Glucose	Monod	0.084	213.60	----	[21]
Glucose	Monod	0.650	11.39	----	[22]
Citrus waste pulp	Monod	0.350	10.69	----	[23]
Glucose	Monod	0.133	3.70	----	[24]
Beet molasses	Monod	0.355	6.65	----	[23]
Soft drinks mixture	Andrews	0.606	65.53	0.029	[25]
Sucrose	Andrews	0.103	30.24	109.8	[26]
Sugar cane juice	Andrews	0.500	0.006	139.7	[15]
Glucose	Andrews	0.088	700	3.730	[27]

μ_max_—the maximum specific growth rate; K_s_—the Monod constant; K_i_—the substrate inhibition constant.

**Table 2:** The estimated kinetics parameters fitted to the Andrews model.

μ=μmax∗S/KS+S+(S2ki)	
Reduced Chi Squared	7.90971 × 10^−6^
Adjusted R-Squared	0.99071
μ_max_ (h^−1^)	0.5086 ± 0.04
K_s_ (g.L^−1^)	47.53789 ± 9.27
K_i_ (g.L^−1^)	181.01639 ± 29.14

**Table 3:** The variation of the yield coefficients at different initial substrate concentrations for the Monod model.

Initial Substrate Concentration (g.L^−1^)	Y_x/s_	Y_p/s_
5	0.267	0.384
10	0.282	0.397
15	0.290	0.446
20	0.278	0.435
25	0.283	0.439
Average	0.280 ± 0.0084	0.420 ± 0.0028

## References

[1] Hasanuzzaman M., Rahim N.A., Hosenuzzaman M., Saidur R., Mahbubul I.M., Rashid M.M. (2012). Energy savings in the combustion based process heating in industrial sector. Renew. Sustain. Energy Rev..

[2] Neto M.R.B., Carvalho P.C.M., Carioca J.O.B., Canafístula F.J.F. (2010). Biogas/photovoltaic hybrid power system for decentralized energy supply of rural areas. Energy Policy.

[3] Nel W.P., Cooper C.J. (2009). Implications of fossil fuel constraints on economic growth and global warming. Energy Policy.

[4] Hoel M., Kverndokk S. (1996). Depletion of fossil fuels and the impacts of global warming. Resour. Energy Econ..

[5] Goldemberg J. (2006). The promise of clean energy. Energy Policy.

[6] Jatoi A.S., Parkash A., Aziz S., Soomro S.A., Shah S.F. (2016). Mathematical modeling for ethanol production from molasses using thermotolerant kluyeromyces marxians. Sci. Int..

[7] Demirbas A. (2007). Progress and recent trends in biofuels. Prog. Energy Combust. Sci..

[8] Alvira P., Tomás-Pejó E., Ballesteros M.J., Negro M.J. (2010). Pretreatment technologies for an efficient bioethanol production process based on enzymatic hydrolysis: A review. Bioresour. Technol..

[9] Zentou H., Abidin Z.Z., Zouanti M., Greetham D. (2015). Effect of operating conditions on molasses fermentation for bioethanol production. Int. J. Appl. Eng. Res..

[10] Gasmalla M.A.A., Yang R., Nikoo M., Man S. (2012). Production of ethanol from sudanese sugar cane molasses and evaluation of its quality. J. Food Process Technol..

[11] Elena P., Gabriela R., Camelia B., Traian H. (2009). Bioethanol production from molasses by different strains of *Saccharomyces cerevisiae*. Ann. Univ. Dunarea JOS Galati Fascicle VI Food Technol..

[12] Periyasamy S., Venkatachalam S., Ramasamy S., Srinivasan V. (2009). Production of bio-ethanol from sugar molasses using *Saccharomyces cerevisiae*. Mod. Appl. Sci..

[13] Dodić J.M., Vučurović D.G., Dodić S.N., Grahovac J.A., Popov S.D., Nedeljković N.M. (2012). Kinetic modelling of batch ethanol production from sugar beet raw juice. Appl. Energy.

[14] Garhyan P., Elnashaie S. (2004). Utilization of mathematical models to investigate the bifurcation and chaotic behavior of ethanol fermentors. Math. Comput. Model..

[15] Oliveira S.C., Oliveira R.C., Tacin M.V., Gattás E.A.L. (2016). Kinetic modeling and optimization of a batch ethanol fermentation process. J. Bioprocess. Biotech..

[16] Monod J. (1950). La technique de culture continue, théorie et applications. Ann. Inst. Pasteur.

[17] Rorke D., Gueguim Kana E.B. (2017). Kinetics of bioethanol production from waste sorghum leaves using *Saccharomyces cerevisiae BY4743*. Fermentation.

[18] Srimachai T., Nuithitikul K., Sompong O., Kongjan P., Panpong K. (2015). Optimization and Kinetic Modeling of Ethanol Production from Oil Palm Frond Juice in Batch Fermentation. Energy Procedia.

[19] Ariyajaroenwong P., Laopaiboon P., Salakkam A., Srinophakun P., Laopaiboon L. (2016). Kinetic models for batch and continuous ethanol fermentation from sweet sorghum juice by yeast immobilized on sweet sorghum stalks. J. Taiwan Inst. Chem. Eng..

[20] Manikandan K., Saravanan V., Viruthagiri T. (2008). Kinetics studies on ethanol production from banana peel waste using mutant strain of *Saccharomyces cerevisiae*. Ind. J. Biotechnol..

[21] Ahmad F., Jameel A.T., Kamarudin M.H., Mel M. (2011). Study of growth kinetic and modeling of ethanol production by Saccharomyces cerevisae. Afr. J. Biotechnol..

[22] Shafaghat H., Najafjour G.D., Rezaei P.S., Sharifzadeh M. (2009). Growth kinetics and ethanol productivity of *Saccharomyces cerevisiae* PTCC 24860 on various carbon sources. World Appl. Sci. J..

[23] Raposo S., Pardão J.M., Diaz I., Lima-Costa M.E. (2009). Kinetic modelling of bioethanol production using agro-industrial by-products. Int. J. Energy Environ..

[24] Singh J., Sharma R. (2015). Growth kinetic and modeling of ethanol production by wilds and mutant *Saccharomyces cerevisiae* MTCC 170. Eur. J. Exp. Biol.

[25] Comelli R.N., Seluy L.G., Isla M.A. (2016). Performance of several *Saccharomyces* strains for the alcoholic fermentation of sugar-sweetened high-strength wastewaters: Comparative analysis and kinetic modelling. New Biotechnol..

[26] Guidini C.Z., Marquez L.D.S., de Almeida Silva H., de Resende M.M., Cardoso V.L., Ribeiro E.J. (2014). Alcoholic fermentation with flocculant *Saccharomyces cerevisiae* in fed-batch process. Appl. Biochem. Biotechnol..

[27] Kostov G., Popova S., Gochev V., Koprinkova-Hristova P., Angelov M., Georgieva A. (2012). Modeling of batch alcohol fermentation with free and immobilized yeasts *Saccharomyces cerevisiae* 46 EVD. Biotechnol. Biotechnol. Equip..

[28] Sluiter A., Hames B., Ruiz R., Scarlata C., Sluiter J., Templeton D. (2006). Determination of sugars, byproducts, and degradation products in liquid fraction process samples. Golden Natl. Renew. Energy Lab..

[29] Lee J.-W., Rodrigues R.C.L.B., Kim H.J., Choi I.-G., Jeffries T.W. (2010). The roles of xylan and lignin in oxalic acid pretreated corncob during separate enzymatic hydrolysis and ethanol fermentation. Bioresour. Technol..

[30] Lee J.-W., Rodrigues R.C.L.B., Jeffries T.W. (2009). Simultaneous saccharification and ethanol fermentation of oxalic acid pretreated corncob assessed with response surface methodology. Bioresour. Technol..

[31] Sen R., Swaminathan T. (2004). Response surface modeling and optimization to elucidate and analyze the effects of inoculum age and size on surfactin production. Biochem. Eng. J..

[32] Kovárová-Kovar K., Egli T. (1998). Growth kinetics of suspended microbial cells: from single-substrate-controlled growth to mixed-substrate kinetics. Microbiol. Mol. Biol. Rev..

